# A roadmap to engineering antiviral natural products synthesis in microbes

**DOI:** 10.1016/j.copbio.2020.07.008

**Published:** 2020-12

**Authors:** Jingbo Ma, Yang Gu, Peng Xu

**Affiliations:** 1 Department of Chemical, Biochemical and Environmental Engineering, University of Maryland, Baltimore County, Baltimore, MD, 21250, USA; 2 Key Laboratory of Carbohydrate Chemistry and Biotechnology, Ministry of Education, Jiangnan University, Wuxi 214122, China

## Abstract

Natural products continue to be the inspirations for us to discover and acquire new drugs. The seemingly unstoppable viruses have kept records high to threaten human health and well-being. The diversity and complexity of natural products (NPs) offer remarkable efficacy and specificity to target viral infection steps and serve as excellent source for antiviral agents. The discovery and production of antiviral NPs remain challenging due to low abundance in their native hosts. Reconstruction of NP biosynthetic pathways in microbes is a promising solution to overcome this limitation. In this review, we surveyed 23 most prominent NPs (from more than 200 antiviral NP candidates) with distinct antiviral mode of actions and summarized the recent metabolic engineering effort to produce these compounds in various microbial hosts. We envision that the scalable and low-cost production of novel antiviral NPs, enabled by metabolic engineering, may light the hope to control and eradicate the deadliest viruses that plague our society and humanity.

## Graphical abstract

**Figure:**
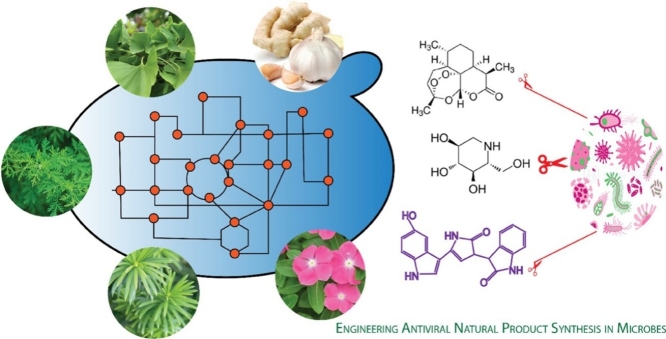

**Current Opinion in Biotechnology** 2020, **66**:140–149

This review comes from a themed issue on **Tissue, cell and pathway engineering**

Edited by **Li Tang**, **Peng Xu** and **Haoran Zhang**

For a complete overview see the Issue and the Editorial

Available online 12th August 2020

**https://doi.org/10.1016/j.copbio.2020.07.008**

0958-1669/© 2020 The Author(s). Published by Elsevier Ltd. This is an open access article under the CC BY license (http://creativecommons.org/licenses/by/4.0/).

## Introduction

Viruses cause numerous human diseases including cancer and severe acute respiratory syndrome (SARS). Millions of people are killed each year due to viral infections, most of which are caused by some notable viruses such as hepatitis B (HBV) and C (HCV) viruses, human immunodeficiency virus (HIV), and influenza virus [1^••^]. The global health is continuously threatened with viral pathogens because of the lack of effective vaccines and drug treatments for many existing and emerging viral diseases. Worse enough, the appearance of immune escape and drug resistant mutants resulted from the high genetic variability of viruses has severely hampered the development of immunological and antiviral therapies [2]. Moreover, due to the rapid urbanization and globalization over the past 20 years, frequent unexpected epidemic outbreaks were caused by novel viruses including severe acute respiratory syndrome coronavirus (SARS-CoV), dengue virus (DENV), Middle East respiratory syndrome coronavirus (MERS-CoV), Ebola virus (EBOV) and SARS-CoV-2 [1^••^,^2^]. Thus, it is of vital importance and urgent advisability to build a full arsenal of novel potent antiviral compounds to combat viral infections and prevent unpredictable global health crises.

Natural products have played a critical role in drug development throughout history. Dated back in 200 AD of the Eastern Han dynasty, the Chinese pharmacologist Dr. Zhang Zhongjing (張仲景 in Chinese) established a number of medical principles to treat infectious disease with Traditional Chinese Medicine. His life-long contribution on medicine has led to the completion of a great medical masterpiece ‘Treatise on Cold Pathogenic and Miscellaneous Diseases’ (傷寒雜病論 in Chinese), which detailed a number of famous herbal prescriptions to treat fever, flu and infectious diseases. With the establishment of chemistry and microbiology as formal disciplines in the 19th century, prominent scientists have discovered antibacterial penicillin from fungi and streptomycin from bacteria, antimalarial artemisinin and anticancer drug paclitaxel from plants [3]. Natural products are obtained by extraction from their native hosts, chemical synthesis or heterologous synthesis. Because of safety and economic concerns, heterologous synthesis in genetically engineered microbes offers some significant advantages over plant extraction and chemical synthesis. With the development of microbial fermentation technology and metabolic engineering strategies, it is possible to engineer cellular biosynthetic machinery and build a scalable and cost-efficient microbial platform to improve the production of various natural products [4]. The advantages of biological production include ambient reaction conditions, flexibility of low-cost and renewable raw materials, ease of large-scale operation, regio-selectivity and stereo-selectivity of biocatalysts to synthesize complex and diverse natural products [4]. This mini-review summarizes the mode of actions of various antiviral natural products and highlights the recent progress in microbial production of these compounds to help advance antiviral drug development. The scalable synthesis of novel antiviral NPs may light the hope to control and eradicate viral infection as well as stop a global pandemic.

## Antiviral mode of actions of natural products

Most viruses possess a limited set of coding genes and must depend on the host machinery to complete viral lifecycle and generate viral progeny [5]. Hence, direct-acting antivirals (DAAs) and host-acting antivirals (HAAs) are two basic mechanisms to develop antiviral agents. One strategy is to interfere with viral DNA replication and protein synthesis by directly acting on viral proteins or genomes, and the other one is to target the common host’s cellular factors involved in viral transmission/propagation or host restriction system such as natural immune response modifiers and components (i.e. interferons) [1^••^]. The HAAs are usually broad-spectrum antivirals (BSAs), which provide several advantages such as treating multiple viruses, higher barrier to the occurrence of resistant viral strains, effective control of new pathogens and reduced drug cross-activity [1^••^,^6^]. The enormous structural diversity and complexity of natural products make them excellent candidates to target-specific biological factors with various mechanisms of action, therefore, natural products continue to be the source of inspiration for novel antiviral drugs [1^••^,^7^]. For example, influenza neuraminidase inhibitors (i.e. oseltamivir sold as *Tamiflu*) are derived from natural product shikimic acid.

**Figure 1:**
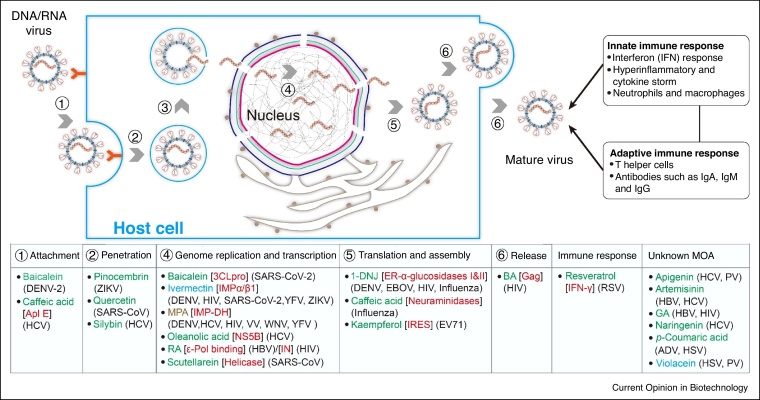
Schematic representation of viral lifecycle and the proposed mode of actions of antiviral natural products discussed in this review. Top: viral lifecycle. The numbers indicate the steps usually targeted by antiviral compounds: 1. viral attachment to host cells, 2. viral penetration into host cells, 3. viral uncoating, 4. viral genome replication and transcription, 5. viral translation and assembly, 6. viral progeny release. Bottom: biological targets of antiviral natural products and their corresponding targeted stages of viral lifecycle. The selected natural products from plants, bacteria and fungi are colored green, blue and brown, respectively. The primary biological targets of antiviral natural products are marked in red color and given in the square brackets. The antiviral spectra of various natural products are also given in the round brackets. MOA, mode of action. Compound abbreviations: BA, betulinic acid; DNJ, 1-deoxynojirimycin; GA, glycyrrhetinic acid; MPA, mycophenolic acid; RA, rosmarinic acid. Molecular target abbreviations: Apl E, apolipoprotein E; 3CLpro, 3C-like protease; ER-α-glucosidases I&II, endoplasmic reticulum α-glucosidases I and II; Gag, HIV Gag protein; IFN‑γ, interferon-gamma; IMPα/β1, importin α and importin β1; IMP-DH, inosine monophosphate dehydrogenase; IN, integrase; IRES, internal ribosome entry site; NS5B, a RNA-dependent RNA polymerase; ε-Pol binding, the interaction between the epsilon (ε) sequence of pregenomic RNA and viral polymerase (Pol). Virus abbreviations: ADV, adenovirus; DENV, dengue virus; DENV-2, dengue virus 2; EBOV, Ebola virus; EV71, enterovirus 71; HBV, hepatitis B virus; HCV, hepatitis C virus; HIV, human immunodeficiency virus; HSV, herpes simplex virus; Influenza, Influenza virus; PV, Poliovirus; RSV, Rous Sarcoma virus; SARS-CoV, severe acute respiratory syndrome coronavirus; SARS-CoV-2, severe acute respiratory syndrome coronavirus 2; VV, vaccinia virus; WNV, West Nile virus; YFV, yellow fever virus; ZIKV, Zika virus.

Viral pathogenesis is characterized as a number of distinct stages involving host–virus interactions, including attachment on cell surface, entry into cell through specific receptors, release of viral DNA/RNAs, viral genome replication and transcription, viral protein translation, virus assembly and repackaging, breakdown of host cell and triggering of host immune response *et al.* (Figure 1). A variety of natural products have been reported to possess potent antiviral activities *in vitro* and *in vivo* [1^••^,^2^,^5^,^8^, ^9^, ^10^]. By surveying about 200 natural products with antiviral activity, we have selected 23 promising natural products with distinct antiviral mode of actions shown in Figure 1. Although the mechanisms of action and molecular targets of some natural products are not completely understood, they can be used in combination with other antiviral agents, and serve as a promising source of antiviral drugs and the basis for the development of new antiviral therapeutics [11]. Among them, the FDA approved antiparasitic drugs hydroxychloroquine, artemisinin and ivermectin were found to have antiviral properties [12]. Some nucleotide analogs are also identified as potent antiviral agents, including spongouridine and spongothymidine extracted from marine sources [1^••^]. Repurposing existing drugs is also a viable strategy to expedite the development of novel antiviral agents [13^•^]. Very recently, baicalein and ivermectin have been tested as potential antiviral drug candidates to treat SARS-CoV-2 which is causing the current worldwide pandemic disease of COVID-19 [14,15]. In the next section, we will summarize the common metabolic engineering strategies that have been implemented to improve antiviral NP production.

## Metabolic engineering strategies for antiviral NP production

**Figure 2:**
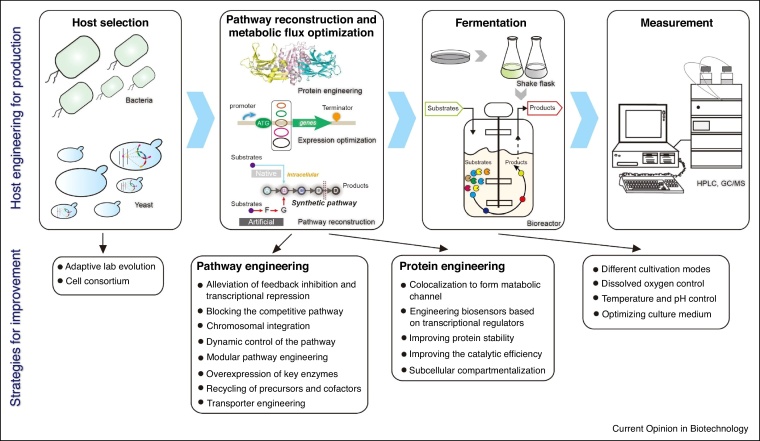
Overview of common strategies used to engineer microbial hosts for improving the production of natural products. In the pathway reconstruction and metabolic flux optimization subpanel, the different circles (from small to large) represent the N-terminal cellular targeting signals or secretion tags, which will direct the protein to different cellular compartment of the cell.

The selection of a suitable microbial host is the first key factor for heterologous pathway expression and antiviral NP production. Chassis engineering offers the advantage to improve the precursor availability and tailor the biochemical reactions inside the cell. *Escherichia coli* and *Saccharomyces cerevisiae* are the two most commonly-used hosts to synthesize natural products. Nonconventional microbes including *Yarrowia lipolytica*, *Rhodosporidium toruloides* and *Pseudomonas putida* are emerging hosts with distinct cellular and metabolic characteristics. The advantages of different hosts have been summarized elsewhere [16]. Adaptive laboratory evolution and microbial consortium can be used to rationally select the desirable phenotype and reduce the host burden to improve the production profile [17]. The development of genetic engineering tools, including gene overexpression, chromosomal gene inactivation and genome evolution, enables us to reconstruct the entire NP biosynthetic pathway in various microbial hosts. Transporter engineering [18], multivariate pathway fine-tuning [19,20], dynamic control of gene expression [21,22] expanded our ability to optimize the flux distribution and construct efficient microbial cell factories. In addition, protein colocalization allows us to engineer enzyme clusters with minimal substrate dissipation and improved catalytic efficiency [23]. And genetically-encoded biosensors may empower us to analyze and monitor cellular process with temporal and spatial resolutions [24]. More importantly, metabolic addiction allows us to link cell growth to an end product that may selectively enrich overproduction subpopulation and improve community-level production performance [25,26^••^]. Bioprocess control with feedback cascades and feeding pattern will be critical to improve the titer and productivity [27]. The commonly-adopted metabolic engineering strategies are outlined in Figure 2, which have been extensively covered in previous reviews [16,17,28, 29, 30]. We will highlight the detailed engineering strategies for the microbial production of specific antiviral natural products in the following section.

## Microbial production of natural products with antiviral activities

We surveyed more than 200 natural products that has been documented and tested for antiviral activity, ranging from aromatics, terpenoids, polyketides, nucleotide analogs and piperidine alkaloids. Microbial engineering has enabled the synthesis of around 23 promising antiviral drug candidates (Table 1). Because of the lipophilic nature, aromatics are the most abundant and largest family of antiviral NPs that have been engineered in microbes, which primarily act on the attachment, penetration, viral RNA translation and repackaging stage of viral infection (Figure 1). Here, we focus on various strategies recently employed to biosynthesize these antiviral natural compounds in different microbial hosts.

**Table 1:** Selection of production of antiviral natural products in genetically engineered microbes

Natural Products	Host	Main strategies	Titer^a^	Scale^b^		Refs
***Aromatics***						
*p*-Coumaric acid	*S. cerevisiae*	Combining both l-Tyr and l-Phe-derived forming routes, supplying the precursor E4P, optimizing carbon distribution	12.5 g/L (Glu)	BR		[31^••^]
Resveratrol	*S. cerevisiae*	Increasing gene copy number, Enhancing P450 activity, precursors supply of malonyl-CoA and phenylalanine	800 mg/L (Glu)	BR		[32]
Resveratrol	*E. coli*	Constitutive expression of 4-coumaroyl-CoA ligase and stilbene synthase under pGAP promoter, expression of ACC to boost malonyl-CoA	2.30 g/L (*p*CA)	FLK		[33]
Naringenin	*Y. lipolytica*	Combination of feedback resistant DAHP synthase, overexpression of ACC1 to enhance malonyl-CoA and peroxisome biogenesis factor pex10 to increase acetyl-CoA	898 mg/L (Glu)	BR		[34^•^]
Taxifolin	*Y. lipolytica*	Modular optimization of chalcone synthase (CHS) and cytochrome P450 reductases (CPR), overexpression of the precursor pathway ARO1 and ACC1	110.5 mg/L (Glu)	FLK		[37^•^]
Apigenin	*E. coli*	Optimization of different gene combinations	30 mg/L (Glu)	FLK		[38]
Baicalein	*E. coli*	Utilization of the promiscuity and the dual specificity of enzymes, optimization of malonyl-CoA availability	23.6 mg/L (Glu)	FLK		[39]
Scutellarein	*E. coli*	Same as baicalein	106.5 mg/L (Glu)	FLK		[39]
Kaempferol	*S. cerevisiae*	Gene screening, eliminating competitive pathway, overexpressing key enzymes	86 mg/L (Glu)	FLK		[40]
Quercetin	*S. cerevisiae*	Gene screening	20.4 mg/L (Glu)	PLT		[41]
Pinocembrin	*E. coli*	Improving ATP and malonyl-CoA supply	165 mg/L (Glu)	FLK		[42]
Caffeic acid	*S. cerevisiae*	Screening different 4HPA3H complexes	289 mg/L (Glu)	FLK		[43]
Rosmarinic acid	*E. coli*	Modular co-culture engineering	172 mg/L (Glu)	FLK		[44^•^]
Silybin	*S. cerevisiae*	Bioproduction and enzymatic catalysis	105 mg/L (Glu)	BR		[45]
Violacein	*Y. lipolytica*	Overexpressing key genes in chassis strain	366 mg/L (Glu)	FLK		[46^•^]
Violacein	*E. coli*	Combinatorial tuning of gene expression with a mutant T7 promoter library	1829 mg/L (Glu)	FLK		[47]
***Terpenoids***						
Betulinic acid	*S. cerevisiae*	Identifying novel P450 enzymes	1.5 g/L (Glu)	BR		[48]
Betulinic acid	*Y. lipolytica*	Using glycerol as carbon sources	26.5 mg/L (Gly)	FLK		[49]
Glycyrrhetinic	*S. cerevisiae*	Enzyme discovery by mining transcriptome	8.8 mg/L (Glu)	BR		[50]
acid	*S. cerevisiae*	Controlling the catalytic property of P450	36.4 mg/L (Glu)	FLK		[51]
Oleanolic acid	*S. cerevisiae*	Introducing a novel reduction system, Rewiring galactose regulatory network	607 mg/L (Glu)	BR		[52]
Artemisinin	*S. cerevisiae*	Bioproduction and chemical conversion	10 g/L (Glu)	BR		[3]
***Others***						
Ivermectin B_1a_	*Streptomyces avermitilis*	Combinatorial biosynthesis and PKS domain-swapping	1.25 g/L (Glu)	FLK		[56]
Avermectin B_1a_	*Streptomyces avermitilis*	Dynamic degradation of triacylglycerol for efficient rerouting of CoA precursors to PKS synthesis	9.31 g/L (Glu)	BR		[57^••^]
MPA	*P. brevicompactum*	Feeding sorbitol and controlling pH	3.26 g/L (Sbt)	BR		[58]
Valinomycin	*E. coli* cell lysates	Cell-free protein synthesis and two-step enzyme cascades in *E. coli* cell lysates	30 mg/L (Glu)	FLK		[59]
DNJ	*S. lavendulae*	Using precursor, analog, metabolism inhibitors as regulators	296 mg/L (Glu)	FLK		[61]
DNJ	*E. coli*	Rational engineering to boost the precursor fructose-6-phosphate	273 mg/L (Glu)	FLK		[62]

Abbreviations: DNJ 1-deoxynojirimycin; E4P erythrose 4-phosphate; 4HPA3H 4-hydroxyphenlacetate 3-hydroxylase; l-Phe L-phenylalanine; l-Tyr L-tyrosine; MPA mycophenolic acid; P450 cytochrome P450 enzyme.

a Carbon sources used for cultivations, Glu, glucose, Gly, glycerol, Sbt, sorbitol, *p*CA, *p*-coumaric acid.

b The scale of fermentation, BR, bioreactor, FLK, shake flask.

### Aromatics-based antiviral natural products

**Figure 3:**
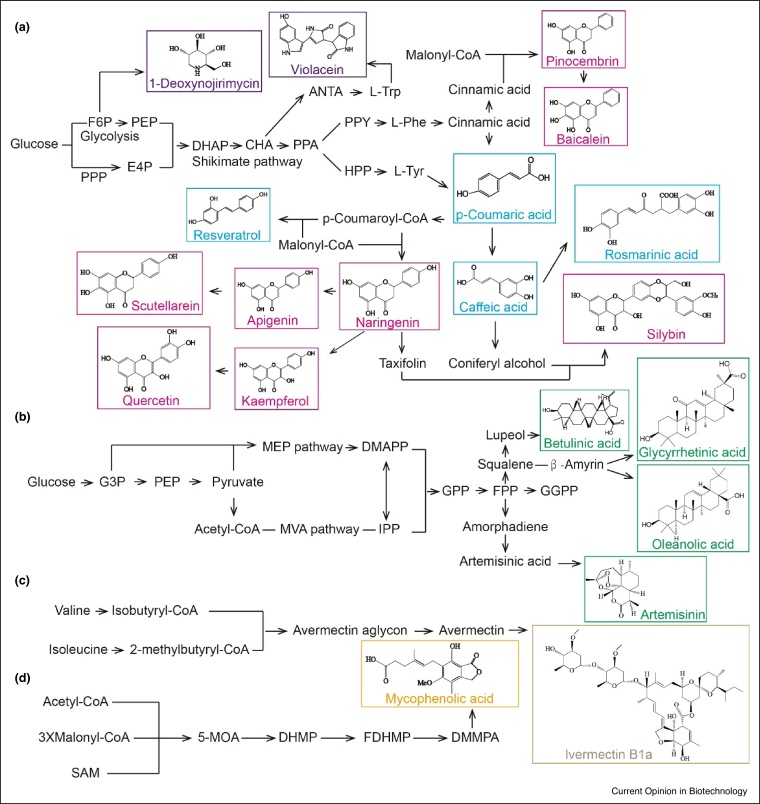
Overview of biosynthetic pathways of antiviral natural products discussed in this review. **(a)** Production of aromatic compounds including flavonoids (magenta), phenol derivatives (indigo), and others (purple). Note, DNJ is not aromatics but belongs to the piperidine alkaloid family. **(b)** Production of terpenoids (green). MEP pathway is used by bacterial hosts, whereas MVA pathway is used by eukaryotes. **(c)** Production of the antibiotic ivermectin B1a (grey). **(d)** Production of the polyketide-terpenoid mycophenolic acid (orange). Abbreviations: ANTA, anthranilate; CHA, chorismate; DAHP, 3-deoxy-d-arabinoheptulosonate 7-phosphate; DHMP, 3,5-dihydroxy-6-methylphthalide; DMAPP, dimethylallyl diphosphate; DMMPA, demethylmycophenolic acid; E4P, erythrose 4-phosphate; F6P, fructose 6-phosphate; FDHMP, 4-farnesyl-3,5-dihydroxy-6-methylphthalide; FPP, farnesyl diphosphate; G3P, glucose 3-phosphate; GGPP, geranylgeranyl pyrophosphate; GPP, geranyl pyrophosphate; HPP, 4-hydroxyphenylpyruvare; IPP, isopentenyl diphosphate; l-Phe, l-phenylalanine; l-Trp, l-tryptophan; l-Tyr, l-tyrosine; MEP, 2-C-methylerythritol-4-phosphate; MVA, mevalonate; 5-MOA, 5-methylorsellinic acid; PEP, phosphoenolpyruvate; PPA, prephenate; PPP, pentose phosphate pathway; PPY, phenylpyruvate; SAM, *S*-adenosyl-l-methionine.

A range of aromatic compounds, including flavonoids and phenylpropanoid derivatives, have been produced through the shikimate pathway, as shown in Figure 3a. *p*-Coumaric acid, an ubiquitous precursor for the production of numerous flavonoids and stilbenoids in plants, is derived from l-tyrosine (l-Tyr) directly generated by tyrosine ammonia lyase (TAL). An alternate route to *p*-Coumaric acid starts with l-phenylalanine (l-Phe) *via* two sequential steps catalyzed by phenylalanine ammonia lyase (PAL) and cinnamate 4-hydroxylase (C4H) with its cytochrome P450 reductase (CPR). A *S. cerevisiae* strain producing 12.5 g/L *p*-coumaric acid was recently constructed by engineering both l-Tyr and l-Phe pathway, recruiting the precursor E4P (erythrose 4-phosphate) with overexpression of a phosphoketolase (PK), and engineering promoters of genes at important nodes connecting glycolysis and shikimic acid pathway [31^••^]. *p*-Coumaroyl-CoA is converted from *p*-coumaric acid by 4-coumarate:CoA ligase (4CL). Then, resveratrol or naringenin can be synthesized by the condensation of *p*-coumaroyl-CoA with three molecules of malonyl-CoA. By optimizing copy number of genes in the resveratrol biosynthetic pathway, enhancing C4H activity, boosting malonyl-CoA supply with the overexpression of a acetyl-CoA carboxylase variant (ACC1^S659A, S1157A^) and increasing phenylalanine flux by the deletion of phenylpyruvate decarboxylase, the engineered *S. cerevisiae* strain finally produced 800 mg/L resveratrol in fed-batch fermentation [32]. Previously, constitutive expression of 4-coumaroyl-CoA ligase and stilbene synthase under pGAP (glyceraldehyde-3-phosphate dehydrogenase) promoter led to the production of 2.3 g/L resveratrol in an optimized *E. coli* culture [33]. Very recently, 898 mg/L naringenin was achieved from a *Yarrowia lipolytica* strain with a *β*-oxidation mediated strategy. Impressively, the naringenin titer experienced a fourfold increase when the authors combined the feedback insensitive mutant of 3-deoxy-d-arabino-heptulosonate-7-phosphate synthase (DAHPS) with the overexpression of the native peroxisome biogenesis factor gene *PEX10* [34^•^].

Taxifolin (dihydroquercetin) is the hydroxylated products of naringenin and has been used to treat viral pancreatitis [35]. By tuning the expression of chalcone synthase, flavanone 3’-hydroxyase and cytochrome P450 reductases, taxifolin production was increased to 110.5 mg/L in *Y. lipolytica* culture [36,37^•^]. Apigenin was synthesized from *p*-coumaric acid in *E. coli* by combinatorial fine-tuning of the expression of four genes 4CL, chalcone synthase (CHS), chalcone isomerase (CHI), and flavone synthase (FNS), which improved the titer of apigenin from 13 mg/L to 30 mg/L [38]. Scutellarein and baicalein have similar structures, differing only in the presence of an additional 4-hydroxy group on the B ring of scutellarein. By recruiting PAL, 4CL, CHS, CHI and FNS from different sources in combination with flavone C-6 hydroxylase (F6H) and its partner CPR, the engineered *E. coli* strain produced 8.5 mg/L baicalein with the supplementation of 0.5 g/L l-Phe in the media [39]. Then, with the aid of the promiscuous F6H and the dual specificity of PAL and 4CL, the same *E. coli* strain was able to synthesize 47.1 mg/L scutellarein with feeding of 0.5 g/L l-Tyr [39]. When malonyl-CoA flux in *E. coli* was improved, the titers of baicalein and scutellarein were further improved to 23.6 mg/L and 106.5 mg/L, respectively.

A *de novo* biosynthetic pathway for kaempferol was reconstructed in *S. cerevisiae* by optimizing the expression of flavonol synthase (FLS) and flavanone 3-hydroxylase (F3H) from a large variety of gene candidates. By blocking phenylethanol accumulation, supplementing intermediates, and overexpressing the key genes in kaempferol synthetic pathway, the final strain produced 86 mg/L kaempferol [40]. In another study, based on production of kaempferol, further introduction of an optimized flavonoid monooxygenase (FMO) and its partner CPR led the production of 20.4 mg/L quercetin in the *S. cerevisiae* strain. Regulation of ATP levels was also found important to improve flavonoid production [41]. For example, pinocembrin titer in *E. coli* was improved by 6.9-fold through transcriptionally silencing two genes *metK* and *proB* involved in regulating ATP level. Further enhancement of malonyl-CoA supply improved pinocembrin production by 1.6-fold with a final titer at 165 mg/L [42] in *E. coli*.

The biosynthetic pathway for caffeic acid was reconstructed in *S. cerevisiae* by introducing tyrosine ammonia lyase (TAL) and different 4-hydroxyphenylacetate 3-hydroxylase (4HPA3H) complexes, and the engineered strain produced 289 mg/L caffeic acid [43]. Rosmarinic acid is condensed from two parallel precursors caffeic acid and salvianic acid A, both of which are derived from l-Tyrosine. This non-linear pathway requires the proper flux balancing for both precursors, which can be solved by modular co-culture engineering. The biosynthetic pathway for rosmarinic acid was partitioned into three modules (caffeic acid module, salvianic acid A module, and rosmarinic acid module) that were engineered in three individual *E. coli* strains. The optimized co-culture system finally yielded 172 mg/L rosmarinic acid, which displayed a 38-fold improvement when compared to the monoculture strategy [44^•^]. The high-value silybin is derived from taxifolin and coniferyl alcohol. To synthesize silybin, two *S. cerevisiae* strains were engineered to produce taxifolin and coniferyl alcohol, respectively. After purification, the two precursors were fed to an *E. coli* cell lysate containing ascorbate peroxidase 1 for silybin synthesis, which achieved a 62.5% yield of silybin [45]. The combination of modular microbial consortia with *in vitro* biotransformation may overcome redox or precursor incompatibility issues for quick access of antiviral natural products.

Violacein, derived from l-tryptophan (l-Trp), is a purple indolocarbazole that is natively produced in some gram-negative bacteria by the *vioABCDE* operon. Its distinct color, broad antibacterial and antiviral activity have made this compound an interesting target for metabolic engineering practices. An *Y. lipolytica* chassis strain was recently engineered by overcoming the rate-limiting steps of the shikimate pathway. By introducing the five genes of *vioABCDE* operon and overexpressing endogenous anthranilate synthase 2 and 3, violacein production was increased to 366 mg/L, representing a 2.9-fold increase compared to the control strain [46^•^]. Combinatorial tuning of the five genes of the violacein pathway with a mutant T7 promoter library led to the production of more than 1.8 g/L of violacein in an optimized *E. coli* culture [47]. These novel compounds with unique structures pave the way for discovery of new antiviral compounds that may cure some deadly viral infections.

### Terpenoids-based antiviral natural products

Terpenoids primarily block viral DNA replication, transcription and mature virus release (Figure 1). They are synthesized from IPP and DMAPP precursors *via* MEP pathway in bacteria or the MVA pathway in yeasts (Figure 3b). Betulinic acid is a lupane-type triterpenoid. Recently, the cytochrome P450 enzyme (CYP) CYP716A155 from the plant rosemary was found to be highly specific to catalyze lupeol to betulinic acid. The engineered *S. cerevisiae* with two copies of CYP716A155 improved the titer of betulinic acid by ninefold from 21.7 mg/L to 193.5 mg/L, yielding a final titer of 1.5 g/L in a 5-L fermenter [48]. The titer of betulinic acid in *Y. lipolytica* was improved by twofold using glycerol as carbon source [49]. Glycyrrhetinic acid, a pentacyclic triterpenoid, is synthesized from *β*-amyrin by the sequential actions of two CYPs, CYP88D6 and CYP72A154. With transcriptome mining, the authors identified cytochrome b5 from *Glycyrrhiza uralensis* and introduced this novel gene into *S. cerevisiae* and improved the glycyrrhetinic acid titer by eightfold [50]. CYP72A63 is another CYP for glycyrrhetinic acid biosynthesis but has poor selectivity. To improve its chemoselectivity and regioselectivity, the promiscuous CYP72A63 was rationally remodeled by mutating key residues identified through computer-aided homology modeling and molecular docking. The glycyrrhetinic acid titer in *S. cerevisiae* finally achieved 36.4 mg/L, which is the highest reported titer in engineered microbes [51]. To achieve high catalytic efficiency between CYP and CPR, four CPRs paired with CYP716A12 were screened for oleanolic acid biosynthesis in *S. cerevisiae*, which improved oleanolic acid production by 2.6-fold. After deleting two genes (*GAL80* and *GAL1*) that are responsible for galactose utilization regulation, gene expression was enhanced and the oleanolic acid titer was improved by 7.8-fold [52]. The complete biosynthetic pathway of artemisinin’s precursor artemisinic acid was previously reconstructed and optimized in *S. cerevisiae*, achieving the production of artemisinic acid with a titer of 25 g/L in bioreactor. Then, artemisinic acid was extracted and transformed to artemisinin by chemical conversion with a 40–45% overall yield [3]. Recently, oleaginous yeast *Y. lipolytica* has been engineered to produce the artemisinin precursor amorphadiene by using a ‘push-and-pull’ strategy to redirect the precursors acetyl-CoA and HMG-CoA [53]. The terpenoids pathway represents a fascinating arsenal for identification and discovery of novel antiviral NPs in the metabolic engineering community.

Apart from the MVA and MEP pathway, nonnative isoprenoid pathways have been constructed by expressing a promiscuous kinase and isopentenyl phosphate kinase (IPK) [54^••^]. This synthetic pathway bypasses the formation of acetoacetyl-CoA and HMG-CoA, and directly generates IPP and DMAPP precursors by phosphorylation of the cheap chemical prenol and isoprenol. Compared to the lengthy MVA or MEP pathway, the two-step isoprenoid pathway offers a number of advantages, including cheap precursors (prenol and isoprenol), less cellular regulation, no involvement of Coenzyme A *et al.* Similarly, an *E. coli* derived hydroxyethylthiazole kinase and a *M. thermautotrophicus* derived isopentenyl phosphate kinase were characterized and used to construct a synthetic IPP pathway for the production of more than 2 g/L geraniol and about 0.6 g/L total monoterpenoids from prenol [55^•^]. This synthetic pathway is decoupled from central carbon metabolism and sustains high IPP and DMAPP flux, which may facilitate the high-yield and cost-efficient production of a diverse range of antiviral isoprenoids.

### Other notable polyketides, nucleotide analogs and piperidine alkaloids

Some polyketides, nucleotide analogs and piperidine alkaloids have been reported to target viral DNA replication, transcription, translation and viral assembly with high specificity (Figure 1 and Table 1). Notably, ivermectin B_1a_ derived from avermectin exhibited low side-effects but the strongest antiparasitic activity among avermectin derivatives, and is the glycosylated product of ivermectin (Figure 3c). One challenge for ivermectin bioproduction is the low compatibility between the native and heterologous polyketide synthase (PKS). To improve the catalytic efficiency, the DH-KR (dehydratase-enoylreductase-ketoreductase) domain of avermectin PKS module II was swapped and replaced with the DH-KR domain of meilingmycin synthase, generating a hybrid gene cluster for ivermectin bioproduction. Effectively, the genetically engineered *Streptomyces avermitilis* was able to produce 1.25 g/L ivermectin B_1a_ [56]. Recently, the intracellular triacylglycerol pool of *Streptomyces avermitilis* was harnessed to dynamically replenish polyketide precursors, leading to the highest avermectin B_1a_ titer at 9.31 g/L in a 180-m^3^ industrial-scale fermenter [57^••^], which forms a basis for commercial production of ivermectin B_1a_.

Mycophenolic acid is a polyketide analog derived from acetyl-CoA, malonyl-CoA and SAM (S-Adenosyl methionine) (Figure 3d), which has been characterized as a first-line immunosuppressant medicine and an RNA capping inhibitor. Bioprocess optimization has led to the production of 3.26 g/L mycophenolic acid in *Penicillium brevicompactum* by feeding sorbitol and controlling the cultivation pH at 6 [58]. Remarkably, a cell-free system has been recently optimized to improve the production of cyclic polyketides valinomycin [59], which has been proven effective to eradicate SARS-CoV virus. Cell free system and synthetic enzyme cascades have also been employed to synthesize the anti-HIV drug islatravir, which is a nucleotide analog and functions as a nucleoside reverse transcriptase translocation inhibitor [60].

1-Deoxynojirimycin (DNJ) is a polyhydroxylated piperidine alkaloid that is derived from fructose-6-phosphate and naturally isolated from mulberry leaves (Figure 3a). Belonging to the heterocyclic nitrogen-containing azasugar family, DNJ is a potent *α*-glucosidase inhibitor with anti-HIV and antitumor activity. The production of DNJ in *Streptomyces lavendulae* was improved by using precursor (glucose), analog (sorbose), and metabolism inhibitors (iodoacetic acid and sodium citrate) as regulators, yielding a titer around 296 mg/L [61]. Recent heterologous expression of DNJ pathway in *E. coli* led to the production of 273 mg/L of DNJ [62], which holds promise for further development. The high titer of polyketides, nucleotide analogs and piperidine alkaloids may facilitate the development of a scalable microbial platform for cost-efficient production of antiviral drugs or prodrugs.

## Conclusions and future perspectives

The global demand of antiviral agents is keeping increasing and nature mother has offered us the solution to treat viral infection with natural products. Plant extraction or chemical synthesis could not meet this demand due to environmental and economic concerns. Microbial fermentation and reconstitution of antiviral NP pathways in microbes provide an alternate for scalable synthesis of these compounds. A wide range of complex natural product derivatives and even unnatural analogues can be generated in microbes by combinatorial biosynthesis and exploiting enzyme promiscuity. Synthetic pathway or novel gene clusters could be discovered by genome mining and engineered for production of novel antiviral NPs. Enzyme catalytic efficiency and specificity could be improved through protein rational design or random mutagenesis. Genome evolution and CRISPR-based tools may offer unprecedented opportunity to expand the existing chemical space and allow us to explore novel antiviral drugs. The convergence of omics-based technologies and synthetic biology makes it more efficient to identify genetic targets and pathway bottlenecks, elucidate missing biosynthetic steps, reconstruct novel and complex biosynthetic pathways, balance metabolic network and optimize metabolic fluxes [63]. Cell-free system offers us the flexibility to debottleneck pathway limitations and quickly access antiviral compounds. Computational approaches and machine learning techniques will be integrated to analyze the large volume of multi-omics datasets, predict and design efficient enzymes and pathways, and largely enhance our ability to screen mutant strains and identify the favorable productive phenotypes as well as accelerate the design-build-test-learn cycle of strain engineering [64, 65, 66]. It is anticipated that microbial metabolic engineering will enter a fascinating era and make significant contributions to antiviral drug discovery and development in the near future.

## Conflicts of interest statement

Nothing declared.

## References and recommended reading

Papers of particular interest, published within the period of review, have been highlighted as:

• of special interest
•• of outstanding interest
